# Drum training induces  long-term plasticity in the cerebellum and connected cortical thickness

**DOI:** 10.1038/s41598-020-65877-2

**Published:** 2020-06-22

**Authors:** Muriel M. K. Bruchhage, Ali Amad, Stephen B. Draper, Jade Seidman, Luis Lacerda, Pedro Luque Laguna, Ruth G. Lowry, James Wheeler, Andrew Robertson, Flavio Dell’Acqua, Marcus S. Smith, Steven C. R. Williams

**Affiliations:** 1 King’s College London, Department of Neuroimaging, Institute of Psychiatry, Psychology and Neuroscience, London, UK; 2 Advanced Baby Imaging Lab, Rhode Island Hospital, 1 Hoppin St, Coro West, Providence, RI USA; 3 Department of Pediatrics, Warren Alpert Medical School at Brown University, 222 Richmond St, Providence, RI USA; 4 Univ. Lille, INSERM U1172, CHU Lille, Centre Lille Neuroscience & Cognition, F-59000 Lille, France; 5 Hartpury University, Hartpury Gloucester, UK; 6 King’s College London, Department of Forensic and Neurodevelopmental Sciences, and the Sackler Institute for Translational Neurodevelopmental Sciences, Institute of Psychiatry, Psychology and Neuroscience, London, UK; 7 University of Chichester, Department of Sport and Exercise Sciences, Chichester, UK; 8 Queen Mary University, Centre for Digital Music, School of Electronic Engineering and Computer Science, London, UK; 9 Developmental Imaging and Biophysics Section, UCL Great Ormond Street Institute of Child Health, London, UK; 10 University of Essex, School of Sport, Rehabilitation and Exercise Sciences, Essex, UK

**Keywords:** Neuroscience, Sensorimotor processing

## Abstract

It is unclear to what extent cerebellar networks show long-term plasticity and accompanied changes in cortical structures. Using drumming as a demanding multimodal motor training, we compared cerebellar lobular volume and white matter microstructure, as well as cortical thickness of 15 healthy non-musicians before and after learning to drum, and 16 age matched novice control participants. After 8 weeks of group drumming instruction, 3 ×30 minutes per week, we observed the cerebellum significantly changing its grey (volume increase of left VIIIa, relative decrease of VIIIb and vermis Crus I volume) and white matter microstructure in the inferior cerebellar peduncle. These plastic cerebellar changes were complemented by changes in cortical thickness (increase in left paracentral, right precuneus and right but not left superior frontal thickness), suggesting an interplay of cerebellar learning with cortical structures enabled through cerebellar pathways.

## Introduction

Early lesion studies by Rolando and Flourens^1^ in the eighteenth century revealed the importance of the cerebellum in motor action and movement coordination, leading Flourens to suggest the cerebellum as a seat for motor learning. Since then, several studies have shown that intense and prolonged motor activity induces significant changes in cerebellar structures. Specifically, motor training in rodent models increases cerebellar glial volume per purkinje cell^2^, volume of the molecular layer^3^, number of synapses^3^ and dendrites of stellate cells^4^. In humans, cerebellar networks show long-term plasticity even into adulthood^5^, indicating experience-dependent adaptive and learning processes to be a salient feature of cerebellar function^6^.

In order for the cerebellum to use plastic processes for skill development, studies suggest that the cerebellum integrates multisensory information in the somatosensory posterior cerebellar lobe to calculate a ‘state estimate’ in order to accurately plan and optimise actions^5,7^, which can be divided into its involvement in sensorimotor and cognitive control functions. This is reached through its broadly distributed system of cortical connections enabled by its three white matter pathways, the cerebellar peduncles^8^. Changes in white matter microstructure in these pathways are indeed often paralleled by alterations in grey matter volume^9,10^. Diffusion tensor imaging (DTI) provides measures to study such changes in white matter pathway microstructure (for specifics^11^) and long-term motor training has been shown to increase white matter fractional anisotropy (FA) of the inferior cerebellar peduncles (ICP^12^). Furthermore, motor training has been shown to increase posterior cerebellar volume and plastic changes in connected cortical regions such as parietal and frontal regions in humans^8^ and animals^13^. Monkey rabies tracer studies revealed that the motor cortex and prefrontal cortex are not only cerebellar output targets. In fact, regions of the cerebellar cortex receiving input from these neocortical areas are the same as those projecting to it and involve different cerebellar regions, with the anterior cerebellum being connected to motor cortices and posterior lateral cerebellar regions to the frontal neocortex^14^. Human functional connectivity resting state analyses support the existence of different, topographically organised loops with sensorimotor or association cortices^15–17^. Specifically, lobule VII has been shown to be functionally connected with frontal and parietal cortices, which are not directly involved in sensorimotor processing^17^, whereas the dorsolateral prefrontal cortex has displayed functional connectivity with lobule VII including Crus I/II, the medial prefrontal cortex with Crus I, and the anterior prefrontal cortex with lobules VI and Crus I/II^16^. These closed-loop circuits represent a fundamental architectural feature of cerebro-cerebellar interactions, which have led many to speculate on whether these neuroanatomic connections in turn impact cerebellar functioning and connectivity. Cerebellar inhibition, by using transcranial brain stimulation, has been shown to abolish plasticity in motor and frontal cortices^8^, enabling its contribution to a broad range of functions from sensorimotor action to higher cognitive function^18^. In order to take this array of cerebellar functions into account, we have used drumming as a coordinated exercise combining musicality, cardiovascular exercise, bilateral arm and leg movements, plus sensory motor integration processes^19^. Moreover, drumming is a unique activity that challenges the brain to synchronize multiple limbs within the constraints of timing, tempo, precision and volume. Here, we chose to use a variety of multimodal MRI measures and analyses: from structural cerebellar segmentation, DTI to cortical thickness.

Using this holistic approach, we expected that drum training would affect cerebellar volume and cortical thickness of both sensorimotor and cognitive control systems in order to meet the high rhythm perception and motor coordination demands to play in time with the music. Due to previous cerebellar findings of decreased functional connectivity in the same group^20^, we specifically expected a decrease in Crus I volume. Finally, we expected that these longer-term plastic changes alter white matter peduncle microstructure as well, as they are the only connection between cerebellar and neocortical structures.

## Materials and Methods

### Participants

Thirty-one right-handed healthy volunteers (16–19 years) with no prior drumming experience and no psychiatric or neurological disorders participated in the study. Informed consent was obtained from all subjects or, if subjects were under 18, consent was obtained from a parent and/or legal guardian. All participants were recruited from the same geographical area and engaged in full time education. To further control for outside influences, we excluded participants engaged in music and dance performance, and asked all participants to continue with their outside school activities as usual. The participants were assigned to one of two groups: the drum group and the control group (Table 1). In order to keep experiences outside the study similar, our volunteers were fellow students matched on age, biological sex and attendance at same education establishment. The King’s College London Research Ethics Committee approved the experimental protocol. All study related procedures were carried out in accordance with the research ethics guidelines outlined in the Declaration of Helsinki.

**Table 1:** Participant demographics displaying participant number, gender and age distribution for both groups (mean and standard deviation; SD).

	Drum group			Control group		
	Male	Female	Total	Male	Female	Total
Number	7	8	15	8	8	16
Mean age in years (SD)	17 (0.58)	16.6 (0.74)	16.8 (0.68)	18 (1.41)	17.8 (1.39)	17.9 (1.36)

### Assessment

All participants attended two scanning sessions at the Institute of Psychiatry Psychology and Neuroscience (IoPPN) at King’s College London. At the first scanning session (T1) the Edinburgh Handedness Inventory Short Form^1^ was used to assess participant hand dominance. To assess the baseline drumming ability and musical experience, a self-report measure was created. Participants were asked to report their level of skill and length of involvement in playing of another musical instrument and (ii) involvement in dance or singing in order to provide a measure of general musical experience to control for skill novelty. Responses were coded on an ordinal scale (0, no experience; 1, some experience but no formal instruction; 2, limited formal instruction but not recent; 3, formal instruction of less than 4 years but not current; 4, formal instruction, exams achieved, greater than 5 years involvement and current).

### Drumming measures

Following the first drumming assessment and scanning session the drum group took part in three 30-minute low intensity group drumming sessions per week for 8 weeks. Each session was delivered by the same professional drum tutor and comprised of 4 integrated parts: (i) a warm up, focused on playing the drums with a relaxed and consistent motion of the drum sticks; (ii) snare drum ‘rudimental’ exercises, played on a single drum surface, adopting a ‘flow sticking’ approach to sequences of left and right hands^21^; (iii) coordinated ‘groove’ patterns, incorporating the interplay of bass drum (right foot) and the hi-hat pedal (left foot) with rock/pop back beat *ostinato* patterns played on the hi-hat or ride cymbal and snare drum; including eighth note (quaver), quarter note (crotchet), sixteenth note (semiquaver), syncopated quarter note and shuffle continuously repeated rhythm; and (iv) performance of learned ‘grooves’ and ‘fill-ins’ to accompany well-known popular music songs. The complexity of drumming tuition was increased on a weekly basis in line with participant’s demonstration of improved drumming coordination and technique. The control participants were asked to not take part in any musical activities for the duration of the study. After the 8 weeks (T2) participants came back to the IoPPN for a second drumming assessment and scanning session.

All of the drumming was performed on electronic drum sets for both drumming training (HD3, Roland, Nakagawa, Japan) and assessment (TD9, Roland, Nakagawa Japan)^21^. Drumming proficiency was assessed following a 5 minute instruction period by the participants’ ability to play a simple 4 quarter note pattern to the song “Green Onions” (Booker T and the MGs, Stax/Atlantic, 1962) and a simple 8 eighth note pattern, consisting of regular eighth note hi-hats with alternating kick and snare on the main beats of the bar, to the song “Billy Jean” (Michael Jackson, EPIC, 1982). Versions without tempo fluctuations were used, created using the software “Live” (V9.1, Ableton, Berlin), creating a precise sample for each beat location, accurately placed on the transient of each audible click^20^ (see supplementary material for more details). Timing data were exported from the drum set using the musical instrument digital interface (MIDI) signal. A comparison with a piezo microphone placed on the snare indicated that the recorded MIDI events were a maximum of 4 ms from the detected onset using audio-based methods, and generally much closer. Drumming ability was assessed objectively as the percentage of bars of both patterns that were completed during two 2-min periods of data capture (1–3 min of each song). To record a completed bar all elements had to be present in each pattern and within half a beat (250 ms for 4 quarter note pattern and 125 ms for an 8 eighth note pattern) of the perfect timing. To evaluate the error in events that should have been synchronous (flamming), a flam error per bar was measured (in milliseconds). The time between the two events was measured for each note of the bar. When 3 limbs were involved, the difference between the first and last event was measured. This was only evaluated in completed bars to avoid exaggeration of this error when the pattern was breaking down or incomplete. While a drum flam can be a sought-after stylistic feature of a pattern, flams were not part of the stylistically correct performance for the two chosen patterns.

For both patterns each limb played a single part of the kit. To perform Green Onions the right hand played ride cymbal, left hand snare drum, right foot kick drum and left foot high-hat pedal. A bar was made up of right hand and right foot striking together followed by right hand, left hand and left foot striking together (repeated to make the four notes of the bar). The Hi-hat, Ride and Snare (HRS) condition required the participants to use their left foot, right hand and left hand. The flam error for this condition was calculated as the time difference (ms) between the first and last action at each point in the bar. The flam error (ms) from the second and fourth beats of the bar was used as a measure of coordination since this involved the coordination of both hands with the left foot. Changes in bars completed across both the more complex eighth note pattern (Billy Jean) and the less complex quarter note pattern (Green Onions) was used to assess whether the participants had learned to drum (% bars completed), whereas the HRS measure determined how precise their drumming was. No practice of either song used in the assessment was included in the 8 weeks drumming training.

### MRI acquisition

All participants were scanned at the Centre for Neuroimaging Sciences, Institute of Psychiatry, London, UK, using a 3-T GE MR750 Dicovery System (General-Electric, Milwaukee, WI). High-resolution structural T1-weighted volumetric images were acquired with full-head coverage, 196 contiguous slices (1.2 mm slice thickness), a 256×256 matrix, and a repetition time/echo time (TR/TE) of 7.3/3 ms (FA = 11°, FOV = 270 mm²). Diffusion Tensor Imaging data was acquired using a spin-echo echo-planar imaging (SE-EPI) sequence providing whole head coverage with the following parameters: TE = 78.5 ms, TR equivalent to 12 RR interval, FOV = 256×256, matrix size of 128×128, 72 slices with a thickness of 2 mm (no gap) making an isotropic voxel of 2.0×2.0×2.0 mm. Diffusion weighting was applied along 60 uniformly distributed directions and with a b-value of 1500 s/mm^2^. Six non-diffusion weighted volumes were also acquired. The acquisition was gated to the cardiac cycle using a peripheral gating device placed on the participants’ forefinger and with a total scan time of approximately 14 minutes. Consistent image quality was ensured by visually inspecting all datasets.

### Cerebellar Segmentation

Prior to analysis, each T1 MP-RAGE was visually inspected to ensure inclusion of only minimal movement artefacts. No scans were discarded. Regional volume of the cerebellum was calculated using the SUIT toolbox (http://www.icn.ucl.ac.uk/motorcontrol/imaging/suit.htm) of the SPM12 software (http://www.fil.ion.ucl.ac.uk/spm/)^21^. This toolbox provides a high-resolution atlas template of the human cerebellum and brainstem that preserves the anatomical detail of cerebellar structures, as well as dedicated procedures to automatically isolate cerebellar structures from the cerebral cortex and to accurately normalise cerebellar structures to this template. Prior to normalisation, the individually created isolation maps were loaded into FSLView (www.fmrib.ox.ac.uk/fsl) where they were visually inspected against the cropped image and hand corrected if necessary. Using the inverse of the resulting normalisation transform, a parcellation of the cerebellum was obtained, based on the probabilistic magnetic resonance atlas of the human cerebellum^22^ provided within the SUIT toolbox. Volumes of interest were then overlaid onto each individual participant’s structural scan and inspected to ensure accurate registration. Parallel to whole brain analyses^23^, how each lobular volume relates to the rest of the cerebellum throughout development may be conceptualised as each volume’s proportion of the total cerebellar volume (TCV). TCV was calculated as the sum of all cerebellar lobules (I-IV, V, VI, Crus I, Crus II, VIIb, VIIIa, VIIIb, IX, X) and regressed out of all lobular volumes. All used cerebellar volumes from hereon were TCV corrected.

### Cortical Thickness Analysis

The FreeSurfer analysis suite version 5.3.0 (http://surfer.nmr.mgh.harvard.edu/) was used to derive models of the cortical surface in each T1-weighted image. These well-validated and fully automated procedures have been extensively described elsewhere^24–29^. In brief, a single filled white matter volume was generated for each hemisphere after intensity normalisation, skull stripping, and image segmentation using a connected components algorithm^24^. Then, measures of cortical thickness were computed as the closest distance from the grey and white matter boundary to the grey matter and cerebrospinal fluid boundary at each vertex on the tessellated surface. The results were inspected and checked for quality following the ENIGMA protocol (www.enigma.ini.usc.edu).

### Diffusion Tensor Imaging

Diffusion data was pre-processed for motion and geometrical distortion correction using ExploreDTI^30^. For each participant the b-matrix was then reoriented to provide a more accurate estimate of diffusion tensor orientations^31^. Diffusion tensor estimation was performed using a non-linear least square fitting method^32^. FA and Mean Diffusivity (MD) maps were generated. Whole brain tractography was performed using all brain voxels with FA ≤ 0.2 as seed region. Streamlines were propagated using an Euler integration^33^ tractography algorithm with a step size of 0.5 mm and with an angular and anisotropy threshold of 45 degrees and FA ≤ 0.2 respectively. Finally, diffusion tensor maps and whole brain tractography were exported to TrackVis version 0.5.2.2^34^ for manual dissection and quantification fibre tracts of the cerebellar tracts.

### Cerebellar Tract Dissections

To define the cerebellar peduncles, a manual two regions-of-interest (ROI) selection method was used^35,36^. First, inclusion and exclusion ROIs were defined on FA maps to define the three cerebellar tracts: the middle cerebellar peduncle (MCP), as well as the left and right ICP and superior cerebellar peduncle (SCP). To extract clean diffusion measurements avoiding crossing and complex white matter regions, each cerebellar peduncle was cut so that only the tract segment between the two ROI was used for quantification. Inclusion ROIs for the ICP were drawn at the medulla oblongata until the level of the dentate; for the MCP a single tract was dissected by selecting the pontine nucleus and before entering the two cerebellar hemispheres; from above the dentate nucleus to the entrance of the tegmentum in the mesencephalon as landmarks for the SCP. Remaining spurious streamlines were removed.

All ROIs were drawn manually on anatomically defined areas on the mean diffusion map for each subject. Manual labelling of ROIs has been the traditional method of analysis for functional and diffusion MRI1. This method has been the default first step for many developmental studies despite potentially large inter-rater variability due to a bias in ROI selection. Given the variability of size and shape of the deep brain areas defining the cerebellar peduncles (i.e. entrance of the tegmentum in the mesencephalon as landmarks for the SCP), manual labelling is advantageous in that it allows placement of regions specific to each individual. In addition, labelling was performed in native space so it requires minimal processing of the original data.

### Statistical analysis

Analyses were performed using SPSS (Version 22, IBM, Chicago, Illinois, USA). Data were tested for normality using the Shapiro-Wilk test. As drumming performance data were not normally distributed, Mann-Whitney U tests, were used to determine any baseline differences. Baseline differences for sex were analysed using the chi-squared test. Drumming improvements were assessed by performing a Mann-Whitney U test on the delta score (post-pre) for % bars completed and HRS error.

As gender may be a factor modulating musical training^37^ and possibly cerebellar volume^38^, all analyses included gender as a covariate. A two-tailed Spearman’s *Rho* correlation analysis between left VIIIa, VIIIb and vermis Crus I volume and the increase in HRS precision following 8 weeks of drum training (T2) as well as individual ANCOVAs run on cerebellar volumes between groups with gender as a covariate were performed. Significance was set at p ≤ 0.05.

## Results

### Changes in drumming performance

Based on a self-report measure, the drum group and the control group presented with no prior drumming experience (all scores ≤2). Moreover, there were no statistical differences between the two groups in terms of self-reported proficiency in other musical modalities (*p* = 0.530) or dancing and singing (*p* = 0.310) and both groups were matched for gender (*p* = 0.724) and age (*p* = 0.105). The drum group improved drumming performance more than controls, (median (1QR) bars completed (47 (34) vs. 13 (23)%, *p* = *0.002)*. There was no difference between the group for bars completed at baseline (*p* = *0.098)*. (A before/after video is available for illustration (see https://vimeo.com/141911618).

In assessing the HRS (flam) error, the drum group showed a trend to increased precision (median delta change 18%), whilst the control group showed a decreased precision (−12%). However, since this measure was highly variable in novice drummers this did not reach statistical significance (*p* = *0.202)*. There was no difference in HRS error between the groups at baseline *(p* = *0.983)*.

### Changes in regional cerebellar volume

**Figure 1:**
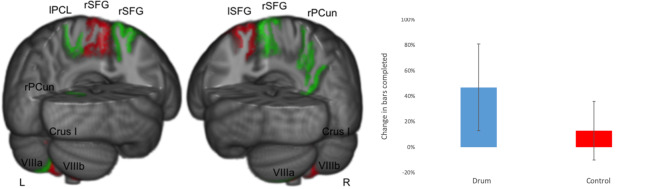
Changes in cerebellar lobule volume and cortical thickness before vs after drum training. Decreasing cerebellar lobules (vermis Crus I, VIIIb) and areas of cortical thickness (left superior frontal cortex, lSFG) in red. Increasing cerebellar lobules (left VIIIa) and areas of cortical thickness (left paracentral lobule, lPCL; right precuneus, rPCun; and right superior frontal gyrus, rSFG) in green. Percentage of bars completed with standard error bars before and after 8 weeks of drum intervention (drum group) and with no intervention (control group) on the right side.

**Figure 2:**
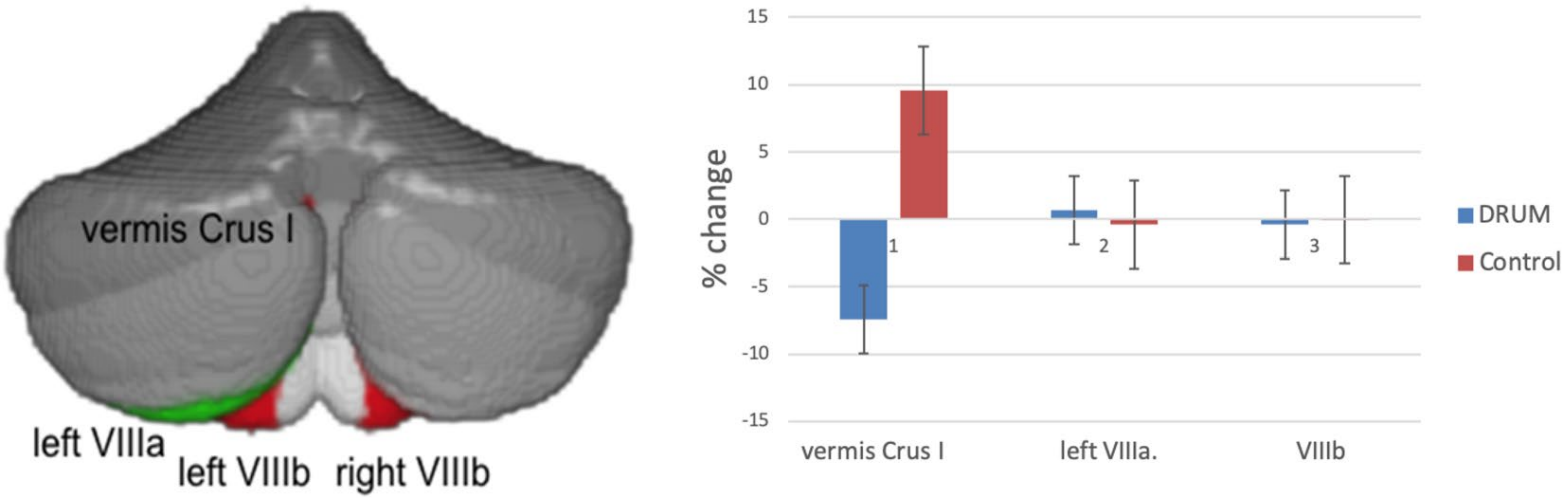
Changes in cerebellar volume increase after drumming. On the left, the increased cerebellar volume is shown in green (left VIIIa), and decreased volume in red (vermis Crus I, left and right VIIIb). On the right, the percentage increase of the vermis Crus I, left VIIIa and VIIIb corrected for total cerebellar volume with standard error bars for the drum and control group are shown (vermis Crus I: drum = −7.4%, control=9.5%; left VIIIa: drum = 0.65%, control = −0.42%; averaged VIIIb: drum = −0.42%, control = −0.03%).

In the drum group, vermis Crus I decreased significantly (corrected model: *F*_*(2,59)*_=4.103, *p* = 0.039; group: *F*_*(1,59)*_=9.250, *p* = 0.012) and lobule VIIIb (left VIIIb: *F*_*(2,59)*_=6.368, *p* = 0.009; right VIIIb: *F*_*(2,59)*_=5.199, *p* = 0.012), whereas the left VIIIa (corrected model: *F*_*(2,59)*_=5.980, *p* = 0.012) increased in volume (Table 2 and Figs. 1, 2). The increase and decrease pattern of the drum group contrasted with the control group can be seen in Table 2, Fig. 1 with scatterplots of differences for each group after drum training in Fig. S1.

**Table 2:** Changes in cerebellar lobular volume (mL) and diffusion measures as well as cortical thickness (mm) after 8 weeks of drum intervention (drum group) and with no intervention (control group), Lobular cerebellar volume of vermis Crus I, left VIIIa, left and right VIIIb were corrected for total cerebellar volume and all analyses included gender and age as covariates.

	Before		After		*p*	*F*
	Control	Drum	Control	Drum		
**Cerebellar grey matter volume (mL)**						
Vermis Crus I	21	27	23	25	0.012*	9.25
Left VIIIa	5743	5367	5719	5402	0.012*	9.16
Left VIIIb	4613	4454	4634	4428	0.003**	11.51
Right VIIIb	4665	4659	4641	4648	0.036*	6.78
**Cerebellar white matter **						
Left ICP						
FA	0.568	0.560	0.565	0.550	0.012*	5.58
MD (x10^-3^ mm^2^/s)	0.709	0.731	0.726	0.722	0.810	1.34
Right ICP						
FA	0.589	0.567	0.580	0.572	0.032*	4.47
MD (x10^-3^ mm^2^/s)	0.698	0.723	0.723	0.707	0.030*	4.54
**Cortical thickness (mm)**						
LPARC	2.43	2.42	2.44	2.45	0.013*	5.10
LSFG	2.84	2.83	2.83	2.83	0.049*	3.39
RPCUN	2.60	2.58	2.63	2.61	0.025*	4.26
RSFG	2.71	2.71	2.74	2.72	0.042*	3.56

Significant changes are marked with ‘*’ for p* ≤* 0.05 and ‘**’ for p ≤ 0.01

Abbreviations: ICP, left inferior cerebellar peduncle; MD, mean diffusivity; FA, fractional anisotropy; LPARC, left paracentral gyrus; LSFG, left superior frontal gyrus; RPCUN, right precuneus; RSFG, right superior frontal gyrus.

**Figure 3:**
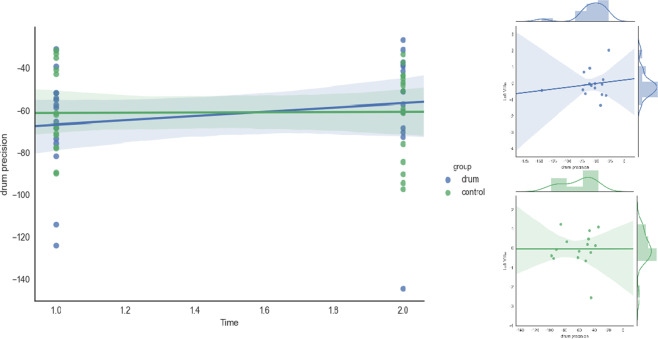
Distribution of drum precision improvement as measured by the High-hat, Ride and Snare (HRS) condition for the drum (blue) and control (green) group on the left and its correlation with left VIIIa volume on the right for each group at the second scanning time point. Confidence intervals are displayed shadowed, individual values as scatterplots (dots) and regression correlation as a continuous line with kernel density fits for each variable on the top and right side.

HRS precision following 8 weeks of drum training (T2) was positively correlated with left VIIIa volume (*τ* = 0.390, *p* = 0.042) after training in the drum group. No significant correlation was evident before drum training (T1) (see Fig. 3).

### Changes in cortical thickness

ANCOVAs were calculated for cortical thickness measures between groups with gender as a covariate revealed a significant volume increase of the left paracentral lobule (lPCL *F*_*(2,59)*_=5.103, *p* = 0.026), the right precuneus (rPCun; *F*_*(2,59)*_=4.264, *p* = 0.050), and the right superior frontal gyrus (RSFG; *F*_*(2,59)*_=3.561, *p* = 0.042), but a decrease of the left superior frontal gyrus (LSFG; *F*_*(2,59)*_=3.385, *p* = 0.049) in the drum group (Table 2, scatterplots of differences for each group after drum training in Fig. S2).

### Changes in white matter microstructure

Individual ANCOVAs were determined for FA and MD in the middle of both hemispheres of the ICP and SCP between groups with gender as a covariate. While FA significantly increased in the right ICP (*F*_*(2,59)*_=4.466, *p* = 0.032) accompanied by decrease in MD (*F*_*(2,59)*_=4.539, *p* = 0.030), the left ICP significantly decreased in FA (*F*_*(2,59)*_=5.583, *p* = 0.012; Table 2, scatterplots of differences for each group after drum training in Fig. S3). Other cerebellar tracts did not show significant differences.

## Discussion

In this longitudinal neuroimaging study, we demonstrate that drum training leads to changes in sensorimotor systems and regions enabling higher cognitive control. Specifically, we show cerebellar volume increases in left VIIIa volume combined with decreases in lobule VIIIb and vermis Crus I. These changes were paralleled with white matter microstructure alterations in the inferior cerebellar peduncle and complemented by increases in prefrontal cortical thickness, suggesting an interplay of cerebellar learning with cortical structures enabled through cerebellar pathways.

### Changes in Sensorimotor Systems

Both clinical observations, cerebellar neuroanatomic connections^39^ and an activation likelihood estimation model^40^ have revealed a dual system of cerebello-cortico interaction and function. This system consists of the ‘sensorimotor cerebellum’ (anterior lobe, lobules VI and VIII) relating to cortical sensorimotor systems and functions, and the ‘cognitive control cerebellum’ (vermis of VI and VII, the hemispheres of lobule VI, Crus I/II and VIIb) connected to prefrontal regions and brain areas involved in higher cognitive functions.

In our plasticity study^20^, we documented changes in several cerebello-cortico sensorimotor regions, including cerebellar lobule VIII, as well as the precentral and paracentral cerebral cortex. A recent meta-analysis of cerebellar functional connectivity clustering cerebellar lobules by behaviour revealed that lobule VIIIa together with VIIb comprise a cluster specialised in action and motor execution^41^. Clinical and behaviour studies have shown that this cluster is further associated with motor processes requiring perceptive feedback and strong attentional control^42^.

Drumming requires both of these functions to help drummers use their arms and legs independently or simultaneously while keeping time with the music, just as in our HRS measure. Here, participants were required to use their left foot, as well as right and left hand to produce a continuous rhythmic pattern. The increase in left VIIIa volume and its positive correlation with error reduction in the HRS measure supports the notion that our training has led to these plastic changes (Fig. 3).

In addition to this volume increase in the cerebellar sensorimotor system, cortical thickness increased in two sensorimotor regions of the neocortex: the precuneus (PCun) and the paracentral lobule (PCL). The precuneus is specialised in translating visuospatial and temporal information into motor coordinates, especially that of sequential movements with reference to memorised patterns^43^. It is further activated during music processing^44^ and can access multiple cerebellar circuits^43^. Functional and structural network studies have described the precuneus as a network hub between parietal and prefrontal regions^45^. Being part of both the prefrontal and parietal cortex, the paracentral lobule innervates motor and sensory modules of the contralateral extremities and its anterior portion is connected to the cerebellum^46^. Cortical thickness has been described as a sensitive measure for plastic changes in the brain^47^ and the action and motor execution cluster including the VIIIa has been previously shown to be co-activated with the precuneus^41^.

### Changes in Cognitive Control Systems

In addition to sensorimotor systems, we also documented changes in cerebello-cortico cognitive control systems, specifically reduced cerebellar vermis Crus I volume and prefrontal thickness in the superior frontal gyrus (SFG). The SFG is important for working memory and executive processing^48^ and it has been suggested that in musicians, deconstructing and organising a rhythm’s temporal structure relates to greater involvement of the prefrontal cortex mediating working memory^49^. Indeed, when participants are required to attend to a motor sequence that they can otherwise perform automatically, an increase in prefrontal cortex activity is observed^50^. Furthermore, lateral posterior regions such as the Crus I have been associated with prefrontal cortical networks^51^, which might suggest a possible connection between the volume decrease of lobule Crus I and LSFG thickness. Paralleling our previous finding of decreased Crus I/ II functional connectivity in the same group^20^, we detected a volume reduction of the vermis Crus I. The cerebellar vermis has been shown to be involved in the production of timed motor responses, and is particularly active when the response is novel^52^ but reduces its activation when a skill has been learned^53^. Furthermore, we suggest that the changes in cortical thickness that we observed cannot be attributed to ongoing neurodevelopmental processes, as pruning reaches its peak in cortical thickness at age twelve in the parietal and prefrontal lobe^54^. Thus, we propose that the improved performance after drum training indicates that a new skill has been learned, with the complex task of drumming creating a need for changes in both sensorimotor and cognitive control systems, as our vast cerebello-cortico volume and thickness changes indicate.

### Microstructural white matter changes in the inferior cerebellar peduncle

To further investigate the linkage between the here documented cerebellar and cortical differences after drum learning, we used diffusion tensor imaging to probe changes in microstructure of the white matter pathways linking the cerebellum to the neocortex. One major white matter pathway highly implicated in plastic changes after skill learning is the inferior cerebellar peduncle^12^.

While the superior cerebellar peduncle represents the main efferent cerebellar output, the middle cerebellar peduncle carries incoming fibres that arise from the pontine nucleus to the opposite hemisphere of the cerebellar cortex, thus enabling communication between both hemispheres^55^. However, the inferior cerebellar peduncle contains mainly afferent pathways carrying sensory information about the body parts to the cerebellum via fibres from the spinocerebellar tract^21^. This enables it to mediate and store a spectrum of motor behaviour^56^, all functions highly necessary for successful drumming. While we documented differences in the main afferent cerebellar pathway, we were unable to show significant differences in white matter microstructure of the superior cerebellar peduncle, its main efferent pathway. Predominant projections to the cerebellum arise from the motor cortex in addition to numerous polysynaptic climbing fibre projections originating from sensory, motor and association areas of the cerebral cortex and are directed to the paravermal and hemispheral cerebellum (see^57^ for review). Thus, our results could indicate that instead of motor cortical-cerebellar modulation, cerebellar multimodal sensory, motor and association projections increasingly influence motor behaviour after being able to drum. As the task has been learned and automatized, it has become implicit, no longer needing explicit neocortical input.

Previous reports have indicated a strong link between changes in structure and diffusion properties, and several studies investigating training effects and brain plasticity have reported increased grey matter volume paired with increased white matter FA^10^ and/or with decreased MD^9^. Interestingly, we were also able to detect such an increase in both FA along with a decrease of MD in the right ICP. These changes in diffusion were accompanied by an increase of left VIIIa volume and a decrease in VIIIb and vermis Crus I volume after training. Interestingly, both the VIIIb and the reticulo-cerebellar tract have been implicated in memory storage related to motor control^58^ and FA and VIIIb volume showed a similar increase-decrease pattern for both hemispheres after drum training (Table 2). However the exact tissue properties of these white matter microstructure changes cannot be determined, as increases in FA can be attributed to several underlying changes in tissue characteristics including an increase in the extent of myelination^11^.

It should be noted, that only the right and not the left SFG increased in cortical thickness after drumming. As drumming is a multi-limb activity, drummers are required to use their four limbs independently or simultaneously when they play, leading to complex inter-hemispheric interactions. It is well known that musical ability is reflected in such left-right differences in brain structure and function^59^, and we were able to display hemispheric differences in functional connectivity in the same group previously^20^. As all of our participants were right-handed, we propose that the MD decrease in the left but not the right ICP paired with the opposite increase-decrease pattern in cortical SFG thickness indicates that left body motor functions have been preferentially strengthened in order to match the high demands of equal velocity needed in drumming. This is further in line with neuroanatomic connections, where the ipsilateral site of the cerebellum has been documented to be active to the body part being moved^40^.

### Future Studies

The complex interplay between the left-right hemispheres, as demonstrated by the results from this and our previous study^20^, should be specifically investigated, for example by using multimodal neuroimaging. Moreover, adding an active control group participating in non-musical motor activities would help distinguish motor action and higher functions involved in music training. Such an active control condition could consist of learning a new physically demanding multi-limb sport (such as playing basketball) or a complex non-rhythmic finger tapping task. To fully understand the extent of the cerebellar learning involved, additional measures that are functionally connected to our results should be applied and more intensive longitudinal scanning comparing both short (e.g. 1 hr, 1 day, 3 days, 1 week) and long-term effects (4, 8 and 16 week) is suggested. Such tasks could be tasks for working memory and emotion regulation, as they have previously been implicated in cerebellar function^18^.

## Conclusion

Drum training acted as a potent stimulus that lead to structural changes in both sensorimotor and cognitive cerebello-cortico systems, possibly leading to the creation of an internal model of drumming patterns. In order to succumb to the high demands of drumming, we propose that the internal model developed in concordance with cortical structures through cerebellar white matter pathways. This way, the brain is able to maintain sustained attention, multi-limb coordination and integrate signals from multiple sensory modalities in order to achieve optimal drum performance. We propose that the increase of cortical thickness reflects an adaptation to the novel demand drumming posits on the brain, namely better temporal accuracy, spatial organisation of movements and strong attentional control enabled by the accumulation of the SFG, PCun and PCL. These structures are further tightly interlinked with each other and the cerebellum, making them an extension of the cerebellar internal model of the drumming rhythms practiced.

## Supplementary Information

Supplementary Information.

Supplementary Information2.
