# Supplementary material for: Drum training induces long-term plasticity in the cerebellum and connected cortical thickness
Source: Sci Rep. 2020 Jun 22;10:10116. doi: 10.1038/s41598-020-65877-2 (PMC7308330; doi:10.1038/s41598-020-65877-2)
Supplement: Supplementary file 1 — Supplementary Information. [file 41598_2020_65877_MOESM1_ESM.docx]

**Drum training induces long-term plasticity in the cerebellum and connected cortical thickness**

**Supplementary Method**

Authors: Muriel M. K. Bruchhage^1,2,3^, Ali Amad^1,4^, Stephen B. Draper^5^, Jade Seidman^1^, Luis Lacerda^9^, Pedro Luque Laguna^6^, Ruth G. Lowry^10^, James Wheeler^7^, Andrew Robertson^8^, Flavio Dell’Acqua^1^ Marcus S. Smith^7†^ and Steven C. R. Williams^1†^*

1. King's College London, Department of Neuroimaging, Institute of Psychiatry, Psychology and Neuroscience, London, UK

2. Advanced Baby Imaging Lab, Women & Infants Hospital of RI, 555 Prospect St, Pawtucket, RI, USA

3. Department of Pediatrics, Warren Alpert Medical School at Brown University, 222 Richmond St, Providence, RI, USA

4. Univ. Lille, INSERM U1172, CHU Lille, Centre Lille Neuroscience & Cognition, F-59000 Lille, France.

5. Hartpury University, Hartpury, Gloucester UK

6. King's College London, Department of Forensic and Neurodevelopmental Sciences, and the Sackler Institute for Translational Neurodevelopmental Sciences, Institute of Psychiatry, Psychology and Neuroscience, London, UK

7. University of Chichester, Department of Sport and Exercise Sciences, Chichester, UK

8. Queen Mary University, Centre for Digital Music, School of Electronic Engineering and Computer Science, London UK

9. Developmental Imaging and Biophysics Section, UCL Great Ormond Street Institute of Child Health, London UK

10. University of Essex, School of Sport, Rehabilitation and Exercise Sciences, Essex, UK

† joint senior authors

*Corresponding author:

Steven C. R. Williams

Centre for Neuroimaging Sciences, Institute of Psychiatry, Psychology & Neuroscience, PO Box 89

De Crespigny Park, London SE5 8AF, UK.

steve.williams@kcl.ac.uk

Phone: +44 (0)20 3228 3060, Fax: +44 (0)20 3228 2116

Main text **4730**words, **60** references, **3** figures, **2** tables

Supplementary Material

*Drumming assessment*

All drumming was performed on electronic drum sets with a standard right-handed 5 piece configuration comprising a snare drum, 3 tom-toms, hi-hat, ride cymbal, crash cymbal, bass drum, and hi-hat pedal (played with the feet)^1^.

Versions without tempo fluctuations were used, created using the software “Live” (V9.1, Ableton, Berlin). This software analysed the beat points of the original recording and adapted them temporally to ensure that the version of the song heard by the participants was at a constant tempo, with a consistent and precise inter beat interval so errors could be accurately recorded. The songs were played out of a single speaker on one channel, while the underlying beat locations were indicated by recording a click track on a second channel. A standard onset detection algorithm ^2^ was used to determine the audio buffer frame in which the transient of each beat in the click track occurred. A precise onset detection method was then used to find the onset location by iteratively dividing the audio buffer into window segments and determining the window in which the energy change was maximal at successively smaller window sizes. Thereby, a precise sample was specified for each beat location, accurately placed on the transient of each audible click^3^.

References

1. Amad, A. *et al.* Motor Learning Induces Plasticity in the Resting Brain-Drumming Up a Connection. *Cereb. Cortex N. Y. N 1991* **27**, 2010–2021 (2017).

2. Albus, J. S. A theory of cerebellar function. *Math. Biosci.* **10**, 25–61 (1971).

3. Robertson, A. Bassline Pitch Prediction for Real-Time Performance Systems. *Int. Comput. Music Conf. Proc.* **2014**, (2014).
