# Supplementary material for: Drum training induces long-term plasticity in the cerebellum and connected cortical thickness
Source: Sci Rep. 2020 Jun 22;10:10116. doi: 10.1038/s41598-020-65877-2 (PMC7308330; doi:10.1038/s41598-020-65877-2)
Supplement: Supplementary file 2 — Supplementary Information2. [file 41598_2020_65877_MOESM2_ESM.docx]

**Drum training induces long-term plasticity in the cerebellum and connected cortical thickness**

Authors: Muriel M. K. Bruchhage^1,2,3^, Ali Amad^1,4^, Stephen B. Draper^5^, Jade Seidman^1^, Luis Lacerda^9^, Pedro Luque Laguna^6^, Ruth G. Lowry^10^, James Wheeler^7^, Andrew Robertson^8^, Flavio Dell’Acqua^1^ Marcus S. Smith^7†^ and Steven C. R. Williams^1†^*

1. King's College London, Department of Neuroimaging, Institute of Psychiatry, Psychology and Neuroscience, London, UK

2. Advanced Baby Imaging Lab, Women & Infants Hospital of RI, 555 Prospect St, Pawtucket, RI, USA

3. Department of Pediatrics, Warren Alpert Medical School at Brown University, 222 Richmond St, Providence, RI, USA

4. Univ. Lille, INSERM U1172, CHU Lille, Centre Lille Neuroscience & Cognition, F-59000 Lille, France.

5. Hartpury University, Hartpury, Gloucester UK

6. King's College London, Department of Forensic and Neurodevelopmental Sciences, and the Sackler Institute for Translational Neurodevelopmental Sciences, Institute of Psychiatry, Psychology and Neuroscience, London, UK

7. University of Chichester, Department of Sport and Exercise Sciences, Chichester, UK

8. Queen Mary University, Centre for Digital Music, School of Electronic Engineering and Computer Science, London UK

9. Developmental Imaging and Biophysics Section, UCL Great Ormond Street Institute of Child Health, London UK

10. University of Essex, School of Sport, Rehabilitation and Exercise Sciences, Essex, UK

† joint senior authors

*Corresponding author:

Steven C. R. Williams

Centre for Neuroimaging Sciences, Institute of Psychiatry, Psychology & Neuroscience, PO Box 89

De Crespigny Park, London SE5 8AF, UK.

steve.williams@kcl.ac.uk

Phone: +44 (0)20 3228 3060, Fax: +44 (0)20 3228 2116

Main text **4730**words, **60** references, **3** figures, **2** tables

**Supplementary Figures**

**Supplementary Figure S1**. Scatterplots of cerebellar lobular volume for the drum and control group after drum training.





**Supplementary Figure S2**. Scatterplots of cortical thickness for the drum and control group after drum training.





**Supplementary Figure S3**. Scatterplots of cerebellar white matter microstructure for the drum and control group after drum training.
